# Probiotic supplementation increases fecal TLR4 agonists without improving disease activity in juvenile idiopathic arthritis: a randomized placebo-controlled trial

**DOI:** 10.1080/19490976.2026.2728351

**Published:** 2026-09-10

**Authors:** Capucine Durand, Laurye-Anne Eveillard, Mathilde Labouret, Florence Aeschlimann, Alexandre Belot, Karine Brochard, Benoit Chassaing, Priscilla Boizeau, Rym Boulkedid, Aurélia Carbasse, Bilade Cherqaoui, Cécile Dumaine, Anne Dumay, Cécile Frachette, Véronique Hentgen, Cécile Kedzia, Therese Kolta, Isabelle Koné-Paut, Quentin Lamy-Besnier, Sophie Guilmin-Crepon, Lara Lima-Antoine, Pascal Pillet, Pierre Quartier, Stéphanie Tellier, Julien Tourneur-Marsille, Heloise Reumaux, Caroline Vinit, Emilie Viennois, Ulrich Meinzer

**Affiliations:** a General Paediatrics, Paediatric Internal Medicine, Rheumatology and Infectious Diseases Department, National Reference Centre for Rare Paediatric Inflammatory Rheumatisms and Systemic Autoimmune diseases (RAISE), Robert-Debré University Hospital, Assistance Publique-Hôpitaux de Paris (APHP), Paris, France; b Université Paris Cité, INSERM, Centre de Recherche sur l’inflammation, Paris, France; c Department of Rheumatology, University Children's Hospital Basel, Basel, Switzerland; d Paediatric Immuno-Haematology and Rheumatology Unit, RAISE Reference Center (ERN RECONNECT), Hopital universitaire Necker-Enfants malades, Paris, France; e Paediatric Nephrology, Rheumatology, Dermatology Department, National Reference Centre for Rare Paediatric Inflammatory Rheumatisms and Systemic Autoimmune diseases (RAISE), Hôpital Femme Mère Enfant, Hospices Civils de Lyon, Bron Cedex, France; f The International Center of Research in Infectiology, Lyon University, Lyon, France; g Paediatric Nephrology and Internal Medicine Unit, Children’s Hospital, Toulouse, France; h Microbiome-Host Interactions, Institut Pasteur, Université Paris Cité, Paris, France; i Assistance Publique–Hôpitaux de Paris (AP-HP), Robert Debré Hospital, Clinical Epidemiology Unit, INSERM Clinical Investigation Center (CIC 1426), Paris, France; j Department of Paediatrics, Arnaud-de-Villeneuve Hospital, Montpellier Regional and University Hospital, Montpellier, France; k Paediatrics Department, AP-HP, Ambroise Paré Hospital, Boulogne-Billancourt, France UMR1173, Université Versailles/Paris-Saclay, INSERM, Infection & Inflammation, Laboratory of Excellence INFIBREX, Montigny-Le-Bretonneux, France; l French Reference Centre for Autoinflammatory Diseases and Amyloidosis CEREMAIA and Competence center for Rare Paediatric Inflammatory Rheumatisms and Systemic Autoimmune diseases RAISE, Department of Paediatrics, Versailles Hospital, Le Chesnay, France; m Clinical Research and Innovation Department, Assistance Publique–Hôpitaux de Paris (AP-HP), Paris, France; n Bicêtre University Hospital (APHP), Paediatric Rheumatology Department and CEREMAIA (Reference Centre for Auto-Inflammatory Diseases and Amyloidosis), Le Kremlin-Bicêtre, Paris, France; o Paediatric Rheumatology Department, National Reference Centre for Rare Paediatric Inflammatory Rheumatisms and Systemic Autoimmune diseases (RAISE), Bordeaux University Hospital, Bordeaux, France; p Paediatric Rheumatology Unit, University of Lille, Jeanne de Flandre Hospital, Lille, France

**Keywords:** Probiotics, gut microbiota, juvenile idiopathic arthritis, innate immunity, Toll-like receptor 4 (TLR4)

## Abstract

Gut dysbiosis has been implicated in the pathogenesis of juvenile idiopathic arthritis (JIA), suggesting that microbiota-targeted interventions may influence immune signalling during early immune development. We conducted the PERMAJI multicentre randomized, double-blind, placebo-controlled trial to evaluate the effects of probiotic supplementation (VSL#3) on host–microbiota immune interactions and disease activity in children with oligoarticular or RF-negative polyarticular JIA. Participants were randomly assigned (1:1) to receive VSL#3 or placebo for 3 months in addition to standard therapy. Stool and serum samples collected at baseline and month 3 were used to assess gut microbiota composition, fecal innate immune agonists, intestinal permeability, and systemic cytokines. The primary clinical endpoint was the proportion achieving an ACR Pedi 30 response at 3 months. Forty-four children were enrolled between September 2017 and July 2022. Clinical responses did not differ between groups (ACR Pedi 30: 47% with VSL#3 vs 63% with placebo; *p* = 0.33), and conservative worst-case assumptions for missing data suggested lower response rates with VSL#3 (36% vs 68%; *p* = 0.03). Probiotic supplementation significantly increased fecal Toll-like receptor 4 (TLR4) agonist activity, whereas gut microbiota diversity, intestinal permeability, and systemic cytokine levels remained unchanged. These findings indicate that probiotic supplementation can modify microbial innate immune signalling without detectable changes in microbial community diversity and may increase exposure to pro-inflammatory microbial stimuli in early-life autoimmune disease. The results highlight the complexity of host–microbiota immune interactions and underscore the need for careful evaluation of microbiome-targeted therapies in paediatric autoimmune disorders.

## Introduction

Juvenile idiopathic arthritis (JIA) is the most common chronic rheumatic disease in children. It affects around 30 out of every 100,000 children in Europe and North America, and is a leading cause of long-term pain, joint damage, and functional disability.^1^ Although disease-modifying antirheumatic drugs (DMARDs) and biological therapies have substantially improved outcomes, a proportion of children continue to experience active disease, relapses, or treatment-related adverse effects, underscoring the need for adjunctive strategies that safely modulate immune dysregulation.

Increasing evidence implicates the intestinal microbiota as a contributor to immune-mediated diseases.^2^ The gut microbiota is established early in life and shaped by diet, antibiotic exposure, environmental factors and host genetics, and plays a role in mucosal barrier integrity and immune homeostasis.^3^ ^,^ ^4^ Disruption to this ecosystem, termed dysbiosis, has been associated with several autoimmune and inflammatory conditions, including JIA.^5^

Observational studies have reported altered gut microbial composition in children with JIA compared with healthy controls, including reduced microbial diversity and shifts in dominant taxa.^6^ ^,^ ^7^ These alterations have been accompanied with signs of intestinal barrier dysfunction. Experimental data further support a contributory role for the microbiota in arthritis pathogenesis: genetically susceptible mice raised under germ-free conditions fail to develop arthritis,^8^ and early-life antibiotic exposure has been associated with an increased risk of JIA in population-based studies.^9^

A proposed mechanistic link between dysbiosis and systemic inflammation is microbial translocation, particularly of lipopolysaccharide (LPS), a component of the cell wall of Gram-negative bacteria.^10^ LPS activates innate immune responses via Toll-like receptor 4 (TLR4), triggering proinflammatory signaling cascades.^11^ Microbial ligands such as LPS represent an important functional interface between the intestinal microbiota and host innate immunity, linking microbial community activity to systemic inflammatory responses. Elevated levels of LPS-related biomarkers have been reported in children with JIA, suggesting increased intestinal permeability and systemic exposure to microbial products,^12^ while animal models indicate that LPS can initiate or exacerbate inflammatory arthritis.^13^

These observations have stimulated interest in microbiota-targeted interventions such as probiotics. Probiotics may influence host immune responses not only through alterations in microbial composition but also through the modulation of microbial-derived immunostimulatory signals. VSL#3 is a multi-strain probiotic formulation with reported immunomodulatory effects and an established safety profile in patients with inflammatory bowel disease.^14-17^ However, evidence for its clinical benefit in children with oligoarticular or RF-negative polyarticular JIA is lacking. Importantly, early childhood represents a critical window of the microbiota,^4^ ^,^ ^18^ characterized by rapid compositional and functional changes that coincide with the maturation of the immune system. Disruptions during this period may have lasting immunological consequences, suggesting that microbiota-directed interventions could be particularly pertinent in young children.^4^ ^,^ ^18^ Experimental murine studies have demonstrated that the presence and composition of the intestinal microbiota are important determinants of arthritis development and severity: germ‑free mice or those treated with antibiotics fail to develop disease in multiple arthritis models, and colonization with specific microbial communities restores susceptibility, indicating a microbiota‑dependent influence on inflammatory joint disease.^8^ ^,^ ^19^ ^,^ ^20^

These observations support biological plausibility for exploring microbiota-targeted interventions in inflammatory joint disease, although direct evidence in JIA is lacking. Despite this biological rationale supported by murine data, no randomized, placebo-controlled trials nor any non-randomized clinical studies have evaluated the clinical or biological effects of probiotic supplementation in recent-onset JIA. In addition, little is known about how probiotic interventions may influence microbial-derived innate immune agonizts that could contribute to systemic inflammation.

We therefore conducted the PERMAJI trial, a multicentre, randomized, placebo-controlled study, to assess the efficacy and safety of VSL#3 as an adjunct to standard therapy in children with children with oligoarticular or RF-negative polyarticular JIA. We hypothesized that probiotic supplementation would modulate the gut microbiota and microbial innate immune signaling, and reduce disease activity.

## Materials and methods

### Study design and participants

This multicentre, randomized, double-blind, placebo-controlled trial was conducted in nine French tertiary care hospitals with pediatric rheumatology expertise. The coordinating center in Robert-Debré Hospital, Paris, oversaw centralized data management and biological analyzes. For microbiota analyzes, all stool samples were centralized, collected, and prepared at the Center de Recherche sur l’Inflammation (INSERM UMR 1149, Paris), and microbiota sequencing and subsequent analyzes were performed at the Institut Pasteur. Patients were recruited across nine French national pediatric rheumatology expert centers, with enrollment per site ranging from 1one to 16 patients. The trial was registered with ClinicalTrials.gov (NCT03092427).

Eligible participants were children aged one to 16 y with oligoarticular or RF-negative polyarticular juvenile idiopathic arthritis (JIA), as defined by ILAR criteria.^21^ The JIA category was assigned by the treating pediatric rheumatologist at study entry. Initially, recruitment was limited to patients with early-onset arthritis, defined as disease onset before the age of 6 y and within 6 months of symptom onset.^22^ Due to low enrollment, eligibility was expanded to include patients with disease onset or clinical exacerbation within the preceding 12 months, with exacerbation being defined as a worsening of disease activity necessitating the initiation or intensification of treatment.

All patients were required to have coverage by the French national health insurance system. Written informed consent was obtained from parents or legal guardians before study procedures commenced. Participant data were coded, securely stored, and accessible only to authorized study personnel in accordance with applicable data-protection regulations.

Exclusion criteria included: systemic JIA; rheumatoid factor positive polyarticular JIA; enthesitis-related arthritis; coexisting autoimmune or autoinflammatory diseases (e.g., inflammatory bowel disease, celia disease, type 1 diabetes, vasculitis, granulomatous disease); history of uveitis >6 months before arthritis onset; obesity (BMI >30 kg/m^2^); significant metabolic, renal, or hematological comorbidities; acute infection or gastrointestinal symptoms within 72 hours of baseline; hypersensitivity to maltose or silicon dioxide; use of lactulose-containing medications; probiotic use within three months; antibiotics within 4 weeks; or planned or ongoing biological treatment during the first three months post enrollment. Post-randomization exclusions included reversal of the JIA diagnosis or development of an ineligible clinical subtype.

No specific dietary restrictions were imposed during the study; participants were allowed to continue their usual diet, and consumption of fermented foods was not considered an exclusion criterion.

Participating centers were requested to report all patients screened for study participation, including those who were not enrolled and the reasons for non-enrollment. However, this information was not available to the coordinating study team, as it was not transmitted by the participating investigators.

### Trial coordination and oversight

The trial was coordinated by Hôpital Robert-Debré, Paris, which was responsible for overall trial coordination and operational management. Scientific and protocol-related decisions were overseen by a steering committee composed of representatives from the Direction de la Recherche Clinique et de l’Innovation (DRCI) and the study coordinators. The steering committee was responsible for oversight of study conduct and for decisions regarding the implementation and management of the protocol. Any important protocol modifications were communicated to the relevant study personnel and participating investigators, and, where required, submitted to the appropriate regulatory and ethics authorities for approval before implementation.

### Sample size and recruitment

The planned sample size was based on detecting a 30% difference in ACR30 response rates between groups with 80% power and *α* = 0·05, yielding 84 patients (42 per group). Recruitment began in September 2017 and continued until July 2022. Due to the COVID-19 pandemic, enrollment was interrupted and did not resume at the anticipated rate. After consultation with the study steering committee, the trial was terminated early after 44 patients had been included.

### Randomization and masking

Patients were randomly assigned in a 1:1 ratio to receive VSL#3 or matching placebo using a centralized web-based randomization system (CleanWeb™). The randomization sequence was generated by the Clinical Research Unit of Robert-Debré Hospital using NQuery® software (Statistical Solutions Ltd.) and was stratified according to two factors: (1) disease activity (oligoarticular JIA: non-existent to low activity [JADAS10 ≤2] versus moderate to high activity [JADAS10 >2]; polyarticular JIA: non-existent to low activity [JADAS10 ≤3.8] versus moderate to high activity [JADAS10 >3.8]) and (2) concomitant JIA treatment (methotrexate and/or NSAIDs). Treatment packs were indistinguishable in appearance, and investigators, participants, caregivers, and outcome assessors remained blinded to treatment allocation throughout the trial. Treatment allocation remained concealed until completion of the primary analyzes.

### Procedures

The probiotic VSL#3 and matching placebo were provided by Actial Pharmaceutical. The intervention was administered orally in powder form, with age-dependent dosing: children aged 1–3 y received 2.25 × 10^11^ colony-forming units (CFU) per day, and children aged 4 y and older received 4.5 × 10^11^ CFU per day. The probiotic was stored and handled according to manufacturer recommendations to preserve bacterial viability.

Participants received either VSL#3 or placebo once daily for 3 months, in addition to standard care. The probiotic and placebo were packaged identically and were indistinguishable in appearance and taste. Participants and care givers and study personnel remained blinded to treatment allocation throughout the study period.

Baseline assessments were conducted at enrollment (month 0 [M0]) and included the collection of demographic and clinical data, as well as blood, urine, and stool samples. Follow-up assessments were performed at month 3. Participants were followed for up to 15 months after enrollment.

Treatment adherence was evaluated using parental reports of missed doses and by counting returned sachets. Adverse events were systematically recorded at each study visit.

### Outcomes

The primary outcome was clinical response at M3 as assessed by the American College of Rheumatology (ACR) Pediatric 30 (ACR30) criteria.^23^ a composite of six variables: i) Number of active joints (with swelling or limitation and pain); ii) Number of joints with limited mobility (excluding irreversibly damaged joints); iii) CHAQ score (range 0–3); iv) Parent or patient global assessment of well-being (100 mm visual analog scale); v) Physician global assessment of disease activity (100 mm visual analog scale); vi) ESR or CRP level.

ACR30 response was defined as improvement in ≥3 of 6 variables by ≥30% from the baseline, with no more than one variable worsening by ≥30% and no new joint infiltration. Participants not meeting these criteria were classified as treatment failures.

Secondary outcomes included ACR50 and ACR70 responses, as well as the JADAS10 score at month 3 (M3). Additional assessments comprised intestinal permeability, measured by the urinary lactulose/mannitol ratio; gut microbiota composition analyzed using 16S rRNA sequencing; and the content of TLR2, TLR4, and TLR5 agonizts in fecal water.

Intestinal permeability was assessed using the lactulose-mannitol test. After oral administration of lactulose and mannitol, urine was collected, and the lactulose/mannitol (L/M) ratio calculated; elevated ratios indicate increased paracellular permeability.

Exploratory subgroup analyzes were performed according to age (<3 vs ≥3 y and <6 vs ≥6 y), concomitant methotrexate use, and baseline disease activity (none/low vs moderate/high). Given the limited sample size and small numbers within individual subgroups, these analyzes were considered exploratory and hypothesis-generating and were not powered for formal subgroup comparisons.

### Gut microbiota analysis

Gut microbiota composition was characterized by 16S rRNA gene sequencing using Illumina technology. DNA was extracted from frozen fecal samples using a Power-feacal pro‑based extraction with mechanical disruption, following the Earth Microbiome Project protocol with modifications, as previously described.^24^ Differences in bacterial community composition were assessed using permutational multivariate analysis of variance (PERMANOVA) implemented with the adonis2 function from the vegan R package on Bray–Curtis distance matrices. The V4 region of the 16S rRNA gene was PCR amplified and sequenced on an Illumina MiSeq platform (paired‑end 2 × 250 bp). Sequencing data were processed with QIIME2 including DADA2 for quality control, phylogenetic diversity was assessed with core‑metrics, and taxonomic assignment was performed against the Greengenes database.^24^ To identify differentially abundant taxa between conditions, we applied Maaslin3 to the QIIME 2-generated genus-level relative abundance table. The analysis used the model ~ Treatment * Timepoint + (1|Individual), with Treatment (VSL#3 vs. placebo), Timepoint (M0 vs. M3), and their interaction specified as fixed effects, and Individual included as a random effect. Statistical significance was defined as an adjusted *p* value < 0.05. All other parameters were set to their default values.

**Fecal load of TLR4, TLR2 and TLR5 agonizts** were quantified using HEK-Blue-hTLR2, HEK-Blue-hTLR4 and HEK-Blue-hTLR5 cells respectively (Invivogen), as previously described.^24^ Fecal supernatants were applied to the cells and incubated for 24 h at 37 °C. Cell culture supernatants were then mixed with QUANTI-Blue medium (InvivoGen), and secreted alkaline phosphatase activity was measured at 630 nm after 30 min. Purified ligands, Pam3CSK4 (InvivoGen), LPS from Escherichia coli (Sigma), and flagellin from *Salmonella typhimurium* (Sigma), were used to generate standard curves. Fecal TLR agonist content was calculated by interpolating sample optical density onto the respective standard curves and is expressed as relative units. Optical density was read at 450 nm using a SPARK 10 M plate reader (Tecan). In figures, values are shown as relative TLR agonist levels derived from the standard curves, without absolute units.

**Serum cytokines** were measured using V-PLEX kits Proinflammatory Panel1 (human, comprising IFNγ, IL-1 *β*, IL-2, IL-4, IL-6, IL-8, IL-10, IL-12p70, IL-13, TNF *α*) TH17 Panel 1 (human, comprising IL-21, IL-31, IL-27, IL-23, IL-22, MIP-3 *α*), U-PLEX kit TGF- *β* 1 and Human/NHP IgG Kit (Meso Scale Discovery, Rockville, MD, USA), according to the manufacturer’s instructions.

**Serum LPS-related markers.** In a post hoc exploratory analysis, stored serum samples were analyzed for functional TLR4 agonist activity, used as a surrogate marker of biologically active LPS, and for anti-LPS IgG antibodies. Functional TLR4 agonist activity was measured using HEK-Blue™ hTLR4 reporter cells (InvivoGen, San Diego, CA, USA). Samples were incubated with the reporter cells according to the manufacturer's instructions, and TLR4 activation was quantified by measuring secreted embryonic alkaline phosphatase (SEAP) activity colorimetrically at 630 nm. Anti-LPS IgG antibodies were measured by enzyme-linked immunosorbent assay (ELISA) using plates coated with Escherichia coli O128 lipopolysaccharide (LPS; Sigma-Aldrich, St. Louis, MO, USA). Bound antibodies were detected using horseradish peroxidase-conjugated anti-human IgG, and optical density was measured at 450 nm using a TECAN Spark 10 M plate reader.

### Statistical analysis

Descriptive statistics were used to summarize baseline characteristics and outcome measures. Qualitative variables are presented as counts and percentages, and quantitative variables as means with standard deviations or medians with interquartile ranges, depending on data distribution.

To determine the optimal judgment criterion, reproducibility analyzes were conducted comparing different versions of the American College of Rheumatology (ACR) response criteria. Agreement between categorical ACR30, ACR50, and ACR70 endpoints using erythrocyte sedimentation rate (ESR) and C-reactive protein (CRP) was assessed using Cohen’s Kappa coefficient with 95% confidence intervals (CIs). Agreement between ESR- and CRP-based versions of the quantitative Juvenile Arthritis Disease Activity Score 10 (JADAS10) was evaluated using the intra-class correlation coefficient (ICC) with 95% CIs. Bland-Altman plots were used to visually assess agreement.

The primary efficacy analysis was conducted according to the intention-to-treat (ITT) principle, including all randomized patients in their originally assigned treatment groups. Categorical outcomes were compared between groups using the Chi-square test or Fisher’s exact test, as appropriate. In the presence of concordance between ACR Pedi 30 assessed erythrocyte sedimentation rate (ESR) and C-reactive protein (CRP), treatment success at month 3 was defined based on ACR Pedi 30 (CRP). Patients who did not meet ACR Pedi 30 improvement criteria were classified as non-responders (failures), including those with stable disease activity.

Missing outcome data for the primary endpoint were handled using three prespecified scenarios: (i) complete-case analysis without imputation; (ii) best-case scenario, in which missing outcomes were imputed as successes in the VSL#3 group and failures in the placebo group; and (iii) worst-case scenario, with missing outcomes imputed as failures in the VSL#3 group and successes in the placebo group. An additional sensitivity analysis was performed in which all missing outcomes were imputed as treatment failures regardless of treatment assignment. These analyzes were considered complementary ITT sensitivity analyzes to assess the robustness of the treatment effect under different assumptions regarding missing data.

Statistical analyzes of stool and serum biomarkers were performed using GraphPad Prism version 9.1.0 (GraphPad Software, San Diego, CA, USA). Data are presented as mean ± standard deviation (SD) or median (interquartile range [IQR]), according to data distribution. The effects of treatment (placebo vs. treatment), time (M0 and M3), and their interaction on biomarker levels were assessed using a two-way repeated-measures ANOVA. When significant main effects or interactions were detected, multiple comparisons were performed using Šidák's post hoc test. Correlations between continuous variables were assessed using Spearman's rank correlation coefficient.

A two-sided *p* value < 0.05 was considered statistically significant. Statistical analyzes were performed using SAS software version 9.4 (SAS Institute Inc., Cary, NC, USA) for clinical outcomes and GraphPad Prism version 9.1.0 (GraphPad Software, San Diego, CA, USA) for stool and serum biomarker analyzes.

## Ethics approval

The study was approved by the national ethics committee CPP Île-de-France VI (study ID 2016-A00727-44). Written informed consent was obtained from parents or legal guardians before any study procedures.

### Trial registration

The trial was registered at ClinicalTrials.gov (NCT03092427).

## Results

### Study population

**Figure 1.**
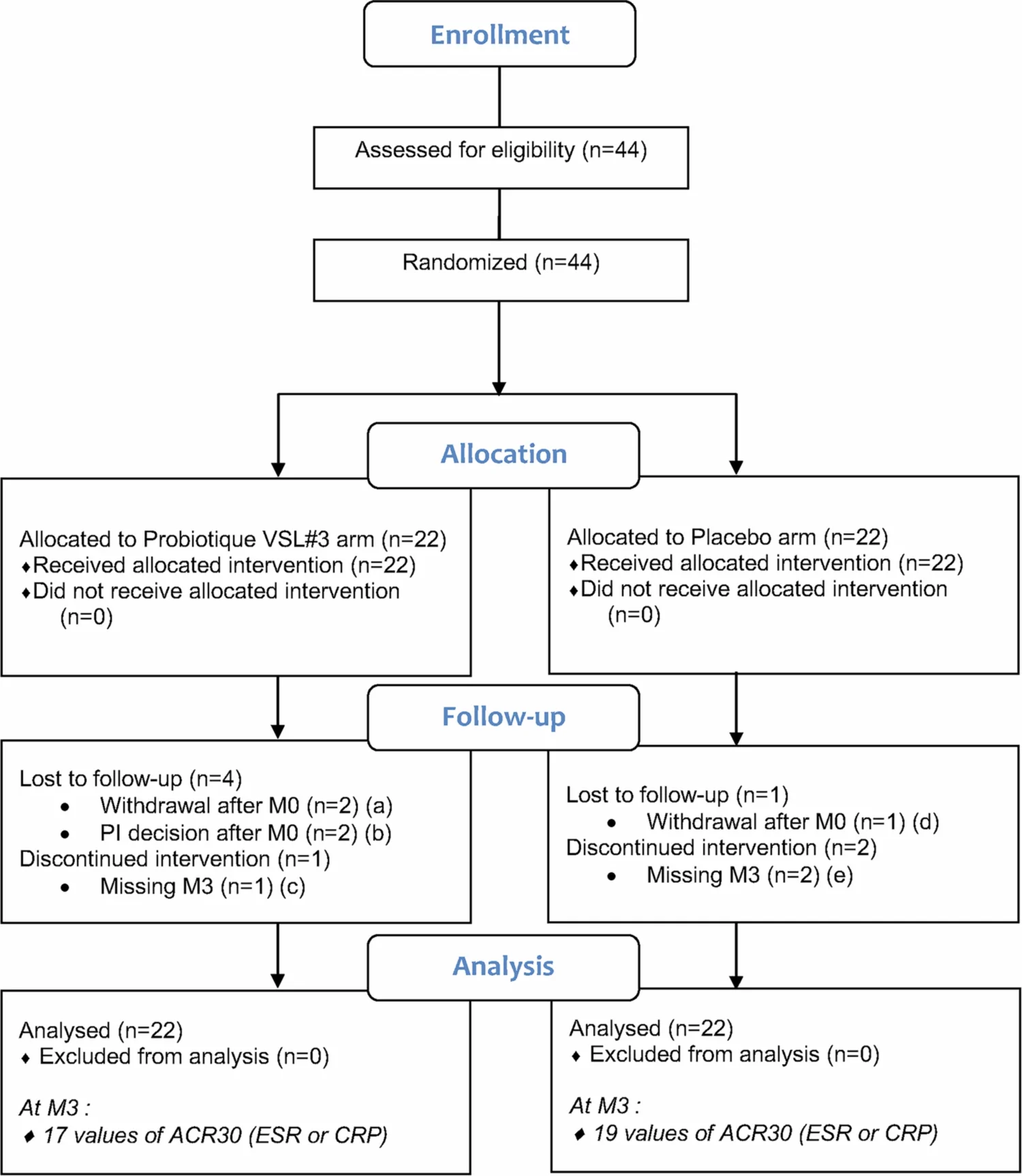
Flowchart of the intention-to-treat population. Details of study withdrawals: (a) Excluded due to difficult or impossible follow-up (*n* = 1) or withdrawal of consent (no longer wished to attend visits; *n* = 1). (b) Excluded due to violation of inclusion criteria (antibiotic intake within 4 weeks prior to inclusion and concomitant biologic therapy; *n* = 1) or initiation of anti-TNF-*α* treatment during follow-up (*n* = 1). (c) Missing Month 3 visit (*n* = 1). (d) Excluded due to difficult or impossible follow-up (*n* = 1). (e) Missing Month 3 visit (*n* = 2). In the additional sensitivity analysis, all patients who withdrew from the study, including the patient who initiated anti-TNF-*α* therapy, were handled using failure imputation (i.e., imputed as non-responders). Abbreviation: PI, Principal investigator.

A total of 44 patients with a diagnosis of juvenile idiopathic arthritis (JIA) were enrolled and randomized to receive either VSL#3 (*n* = 22) or placebo (*n* = 22). All enrolled patients were classified by the investigators as having oligoarticular or polyarticular JIA at study entry, and no patient had psoriasis reported as a concomitant condition. Seven patients (16%) were lost to follow-up: five in the VSL#3 group and two in the placebo group. Reasons for withdrawal included logistical difficulties, patient or caregiver decision, and meeting exclusion criteria (such as recent antibiotic use or initiation of biological therapy). A detailed breakdown of patient attrition is provided in Figure 1.

### Baseline characteristics

Baseline demographic and clinical characteristics were similar between groups (Table 1). The median age was 4.0 (range: 2.8–6.5) years, with a predominance of female patients. Most patients (*n* = 39, 89%) had oligoarticular JIA, and 30 (68%) had absent or low disease activity at baseline. The number of active joints ranged from 0 to 10, with a median disease duration of 5.9 months.

**Table 1.** Baseline demographic and clinical characteristics of patients with juvenile idiopathic arthritis at study inclusion.

Variable	Placebo *N* = 22	Probiotic VSL#3 *N* = 22
**Age at randomization**, in years		
Median (Q1; Q3)	3,9 (2,8; 5,3)	4,7 (3,0; 6,5)
**Age at disease onset**, in years		
Médiane (Q1; Q3)	3,4 (1,8;5,1)	4,2 (2,4;6,4)
**Disease duration at inclusion**, in months		
Median (Q1; Q3)	5,9 (4,1; 7,7)	5,9 (3,8; 7,9)
**Sex**		
Male	8 (36%)	6 (27%)
Female	14 (64%)	16 (73%)
**Ongoing treatment**		
Methotrexate (with or without NSAID)	7 (32%)	7 (32%)
NSAID	15 (68%)	15 (68%)
**Number of inflammatory joints**		
Median (Q1; Q3)	1,0 (0,0; 2,0)	1,0 (0,0; 2,0)
**Number of inflammatory joints**		
0	9 (41%)	10 (45%)
1	7 (32%)	6 (27%)
2	3 (14%)	2 (9%)
3	0 (0%)	2 (9%)
4	1 (5%)	2 (9%)
**≥**5	2 (10%)	0 (0%)
**JIA subgroup**		
Oligoarticular	20 (91%)	19 (86%)
Polyarticular	2 (9%)	3 (14%)
**Disease activity in patients with polyarticular disease**		
None to low disease activity (JADAS10 ≤ 3.8)	1 (50%)	1 (33%)
Moderate to high disease activity (JADAS10 > 3.8)	1 (50%)	2 (67%)
**Disease activity in patients with oligoarticular disease**		
None to low disease activity (JADAS10 ≤ 2)	14 (70%)	14 (74%)
Moderate to high disease activity (JADAS10 > 2)	6 (30%)	5 (26%)
**Follow-up duration, in months**		
Median (Q1; Q3)	11,9 (11,7; 12,0)	12,0 (11,7; 12,2)
**Bristol Stool Scale**		
Median (Q1; Q3)	2 (2; 3)	3 (2; 3)

Approximately one third of participation in each group were receiving methotrexate in addition to and non-steroidal anti-inflammatory drugs (NSAIDs), while the remaining two thirds were treated with NSAIDs alone. Detailed baseline characteristics are shown in Table 1.

Stool consistency assessed using the Bristol Stool Scale did not differ significantly between groups (median [IQR]: 2 [2–3] vs 3 [2–3], *p* = 0.43). A detailed overview of changes in the Bristol Stool Scale, NSAID and methotrexate use, and the occurrence of joint injections over the study period is provided in Supplementary Table 1.

### Safety

Adverse events were reported in both treatment groups during the study period. All adverse events are summarized in Supplementary Table 2.

### Primary endpoint

**Figure 2.**
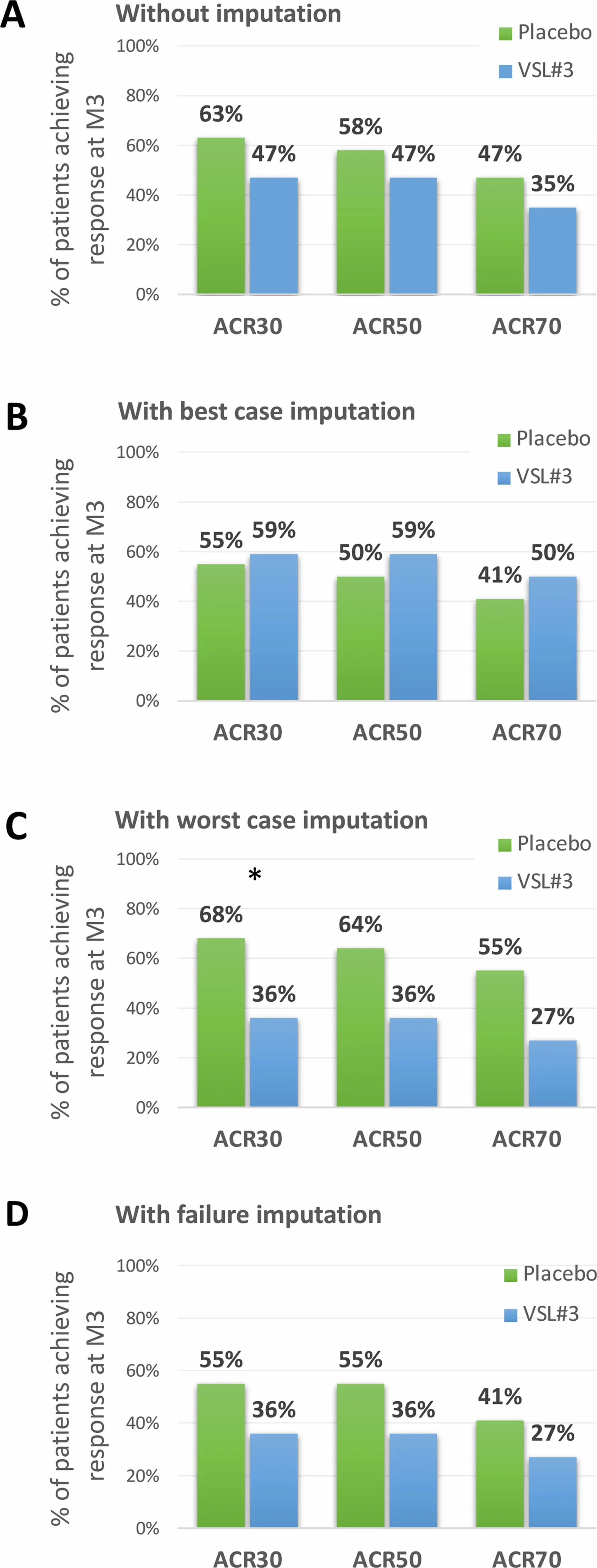
American College of Rheumatology response rates at month 3 in the intention-to-treat population. Bar charts showing the proportion of patients achieving ACR30 (primary endpoint), ACR50, and ACR70 responses at month A in the intention-to-treat (ITT) population: (A) without imputation, (B) with best-case imputation, (C) with worst-case imputation, and (D) with failure imputation. Abbreviations: ACR30/50/70, ≥30%, ≥50%, and ≥70% improvement according to American College of Rheumatology criteria; ITT, intention-to-treat.

At month 3 (M3), response rates for the primary endpoint (ACR Pedi 30) varied according to the prespecified ITT assumptions regarding missing data. In the complete-case analysis, an ACR Pedi 30 response was observed in 8 of 17 patients (47%) in the VSL#3 group and 12 of 19 patients (63%) in the placebo group (*p* = 0.33). In the best-case imputation scenario, response rates were 59% in the VSL#3 group and 55% in the placebo group (*p* = 0.76). In contrast, under the worst-case imputation scenario, response rates were 36% and 68%, respectively (*p* = 0.03; Figure 2 and Table 2). Results of the failure-imputation analysis were consistent with the primary analyzes (Figure 2 and Table 2).

**Table 2.** American College of Rheumatology response at month 3 in the intention-to-treat population.

	Placebo *N* = 22	VSL#3 *N* = 22	*p*-value
**ACR 30 - Primary endpoint**			
30% improvement without imputation at M3	12/19 (63%)	8/17 (47%)	*p* = 0.33
30% improvement with *best case* imputation at M3	12/22 (55%)	13/22 (59%)	*p* = 0.76
30% improvement with *worst case* imputation at M3	15/22 (68%)	8/22 (36%)	***p* = 0.03**
30% improvement with *failure* imputation at M3	12/22 (55%)	8/22 (36%)	*p* = 0.22
**ACR 50 - Secondary endpoint**			
50% improvement without imputation at M3	11/19 (50%)	8/17 (47%)	*p* = 0.52
50% improvement with *best case* imputation at M3	11/22 (55%)	13/22 (59%)	*p* = 0.54
50% improvement with *worst case* imputation at M3	14/22 (68%)	8/22 (36%)	*p* = 0.07
50% improvement with *failure* imputation at M3	11/22 (55%)	8/22 (36%)	*p* = 0.36
**ACR70 - Secondary endpoint**			
70% improvement without imputation at M3	9/19 (47%)	6/17 (35%)	*p* = 0.46
70% improvement with *best case* imputation at M3	9/22 (41%)	11/22(50%)	*p* = 0.54
70% improvement with *worst case* imputation at M3	12/22 (55%)	6/22 (27%)	*p* = 0.07
70% improvement with *failure* imputation at M3	9/22 (41%)	6/22 (27%)	*p* = 0.34

Proportion of patients achieving ACR30 (primary endpoint), ACR50, and ACR70 (secondary endpoints) at month 3 in the intention-to-treat (ITT) population. Response rates are presented without imputation, with best-case imputation, and with worst-case imputation. Comparisons between groups were performed using a χ^2^ (chi-square) test. Data are shown as number of responders/number of evaluable patients (%).

Abbreviations: ACR30/50/70, ≥30%, ≥50%, and ≥70% improvement according to American College of Rheumatology criteria; ITT, intention-to-treat.

### Secondary endpoints

No statistically significant differences were observed between groups for ACR Pedi 50 or ACR Pedi 70 responses (Figure 2 and Table 2). Median JADAS scores showed minimal change at M3 in both groups, with a decrease of 0.1 (IQR –0.2 to 0.2) in the placebo group and no change (0.0 (IQR –0.2 to 0.2) in the VSL#3 group (*p* = 0.85; Table 3). Physician global assessment of disease activity increased by a median of 5 (IQR 0 to 10) points in the VSL#3 group, while it decreased by 5 points (IQR –15 to 0) in the placebo group; however, this difference was not statistically significant (*p* = 0.15; Table 3).

**Table 3.** Evolution of American College of Rheumatology core set variables between baseline (M0) and month 3 (M3).

	Probiotic arm VSL#3 (*N* = 22)			Placebo arm (*N* = 22)		
Variable	M0 (*N* = 22)	M3 (*N* = 18)	Delta M3-M0 (*N* = 18)	M0 (*N* = 22)	M3 (*N* = 17)	Delta M3-M0 (*N* = 17)
Well-being VAS (patient or parents)	*N* = 22	*N* = 17	*N* = 17	*N* = 21	*N* = 17	*N* = 17
Median (Q1; Q3)	10,0 (5,0; 20,0)	10,0 (5,0; 15,0)	0,0 (−10,0; 5,0)	10,0 (5,0; 25,0)	10,0 (0,0; 20,0)	0,0 (−10,0; 4,0)
Disease activity VAS (physician)	*N* = 22	*N* = 17	*N* = 17	*N* = 22	*N* = 17	*N* = 17
Median (Q1; Q3)	12,5 (0,0; 25,0)	15,0 (0,0; 30,0)	5,0 (0,0; 10,0)	10,0 (0,0; 25,0)	5,0 (0,0; 10,0)	−5,0 (−15,0; 0,0)
JADAS 10 (CRP or ESR)	*N* = 22	*N* = 17	*N* = 17	*N* = 21	*N* = 17	*N* = 17
Median (Q1; Q3)	0,4 (0,1; 0,6)	0,4 (0,1; 0,6)	0,0 (−0,2; 0,2)	0,3 (0,2; 0,6)	0,3 (0,1; 0,3)	−0,1 (−0,2; 0,2)
Functionnal score CHAQ	*N* = 21	*N* = 17	*N* = 17	*N* = 21	*N* = 17	*N* = 17
Median (Q1; Q3)	0,4 (0,3; 0,7)	0,5 (0,3; 0,8)	0,0 (−0,3; 0,2)	0,5 (0,3; 0,8)	0,4 (0,3; 0,6)	−0,1 (−0,3; 0,0)
ESR (at 1 hour) in mm	*N* = 21	*N* = 14	*N* = 14	*N* = 20	*N* = 14	*N* = 13
Median (Q1; Q3)	10,0 (5,0; 15,0)	9,0 (5,0; 12,0)	−1,0 (−5,0; 3,0)	15,5 (9,0; 17,5)	11,0 (7,0; 17,0)	0,0 (−8,0; 1,0)
CRP in mg/L	*N* = 22	*N* = 17	*N* = 17	*N* = 22	*N* = 17	*N* = 17
Median (Q1; Q3)	5,0 (4,0; 10,0)	5,0 (4,0; 9,0)	0,0 (−1,0; 0,0)	7,0 (1,1; 10,0)	5,7 (1,0; 10,0)	0,0 (0,0; 0,3)
Number of active joints	*N* = 22	*N* = 17	*N* = 17	*N* = 22	*N* = 17	*N* = 17
Median (Q1; Q3)	1,0 (0,0; 2,0)	1,0 (0,0; 1,0)	0,0 (0,0; 0,0)	1,0 (0,0; 2,0)	0,0 (0,0; 1,0)	0,0 (−1,0; 1,0)

Individual parameters used for the calculation of the ACR30 response are shown at baseline (M0), month 3 (primary endpoint), and as change from baseline (M3–M0) in the probiotic (VSL#3) and placebo arms. Analyzes of change from baseline include only patients with available data at both timepoints. Continuous variables are presented as mean (SD) or median (IQR), as appropriate. When ESR-based JADAS10 values were unavailable, CRP-based JADAS10 values were used.

Abbreviations: ACR, American College of Rheumatology; JADAS, Juvenile Arthritis Disease Activity Score; M0, baseline; M3, month 3; ESR, erythrocyte sedimentation rate; CRP, C-reactive protein; SD, standard deviation; IQR, interquartile range.

### Exploratory subgroup analyzes

Exploratory subgroup analyzes according to age (<3 vs ≥3 y and <6 vs ≥6 y), concomitant methotrexate use, and baseline disease activity (none/low vs moderate/high) showed no consistent pattern of treatment response across subgroups (Supplementary Tables 3 and 4). Although isolated statistically significant differences favouring placebo were observed under the worst-case imputation analysis in the low disease activity and NSAID-only subgroups, these findings were not reproduced across the other imputation strategies. Given the limited sample size, these analyzes should be considered exploratory and hypothesis-generating.

### Gut microbiota analysis stratified by treatment arm and ACR30 response

**Figure 3.**
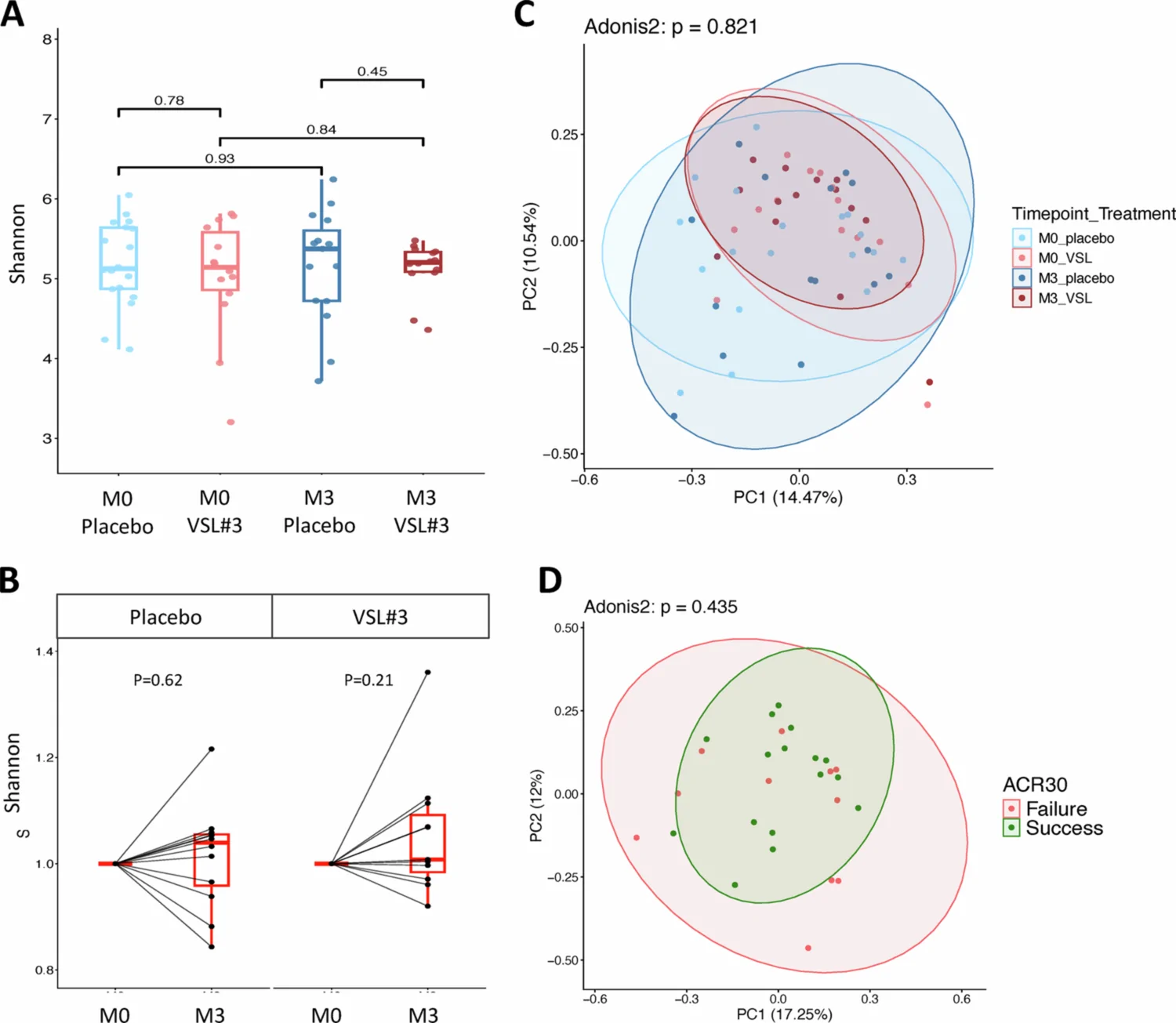
Gut Microbiota Analysis stratified by Treatment Arm and ACR30 response. (A) Alpha diversity (Shannon index) of stool microbiota at baseline (M0) and month 3 (M3) according to treatment received. (B) Fold change in Shannon alpha diversity between M0 and M3 in stool samples by treatment arm. (C) Beta diversity (Bray–Curtis distance) of stool microbiota at M3 according to treatment received. (D) Association between disease activity (ACR30 response) and beta diversity (Bray–Curtis distance) in stool samples at M3. Ellipses represent 95% confidence intervals. The *p* value corresponds to a PERMANOVA test performed using the adonis2 function from the vegan R package. Abbreviations: ACR30, ≥30% improvement according to American College of Rheumatology criteria; M0, baseline; M3, month 3.

Gut microbiota composition did not differ between groups at baseline nor after probiotics intervention. Specifically, alpha diversity, measured by the Shannon index, was similar between the probiotic (VSL#3) and placebo groups at baseline (M0) and remained unchanged after 3 months of treatment (Figure 3A). Moreover, fold changes in such alpha diversity metrics of stool samples over the 3-month period were not significantly affected by treatment assignment (Figure 3B). Beta diversity analysis using principal coordinate analysis (PCoA) also showed no differences between groups at baseline and no significant shifts after the intervention period (Figure 3C). Additionally, there were no differences in the gut microbiota community at 3 months (M3) based on disease activity (ACR30) (Figure 3D). Differential abundance analysis of bacterial genera represented in VSL#3 did not identify significant treatment-related increases in any probiotic-associated genus (Supplementary Figure S3).

### Stool biomarkers

**Figure 4.**
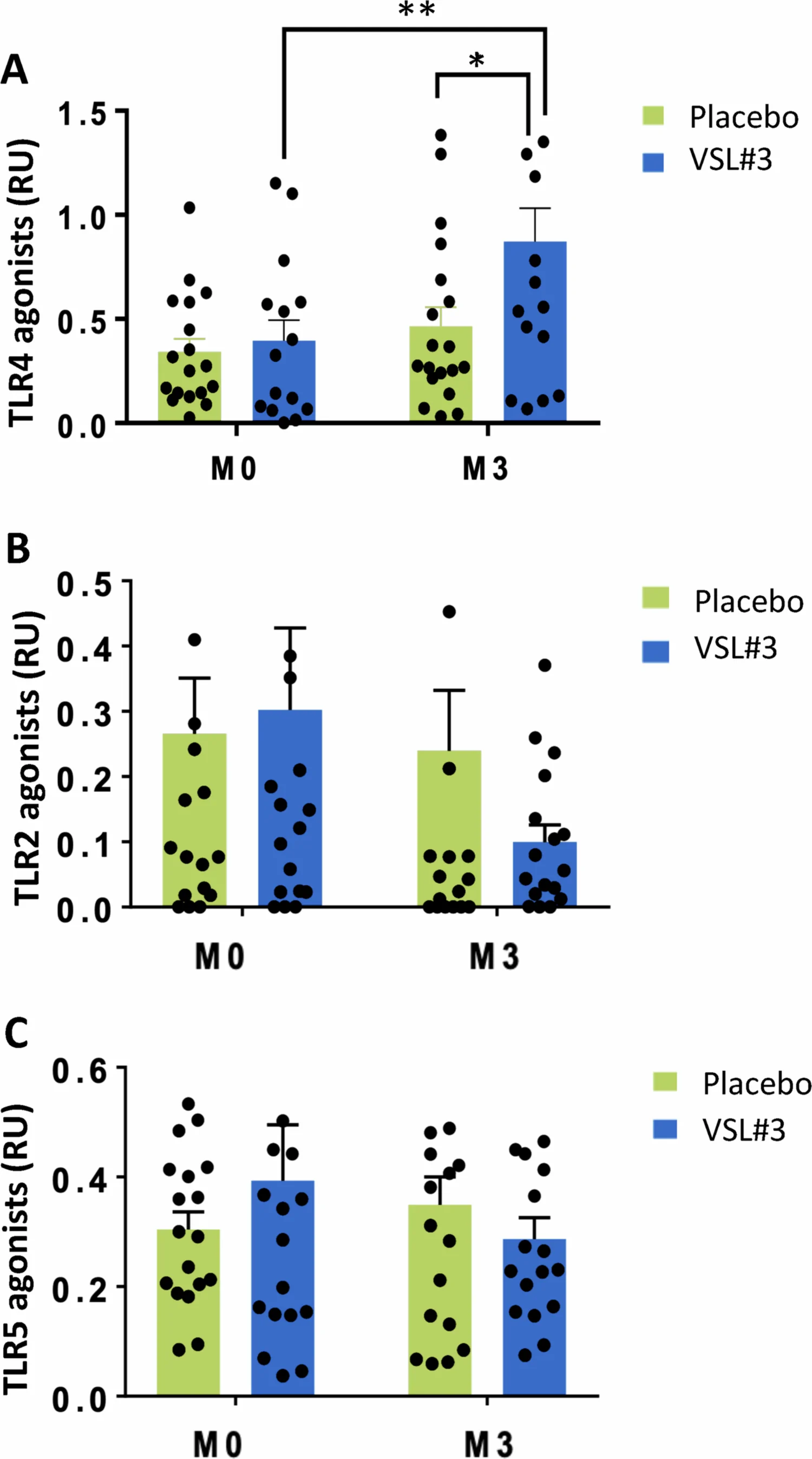
Quantification of fecal TLR agonist activity in stool samples before and after treatment. Fecal TLR4, TLR5, and TLR2 agonist activity in stool samples at baseline (M0) and month 3 (M3) in patients treated with VSL#3 or placebo. Panels show: (A) TLR4 agonizts, (B) TLR5 agonizts, and (C) TLR2 agonizts in fecal water. Fecal TLR agonist levels were determined using HEK-Blue reporter cells and are expressed as relative units (RU) derived from standard curves generated with the corresponding purified ligands (lipopolysaccharide [LPS], Pam3CSK4, or flagellin). Sample size *n* = 15 to 20 per group. Data are presented as mean ± SD. Statistical analysis was performed using two-way ANOVA followed by Šídák's multiple comparisons test. *P = 0.0215 and **P < 0.0063. Abbreviations: TLR, Toll-like receptor; M0, baseline; M3, month 3; SD, standard deviation; RU, relative units; LPS, lipopolysaccharide.

Fecal levels of TLR4 agonizts (lipopolysaccharide, LPS) increased significantly from baseline to M3 in the VSL#3 group (*p* < 0·01; Figure 4A), whereas no significant changes were observed in the placebo group. At M3, fecal levels of TLR4 agonizts were higher in patients treated with VSL#3 than those receiving placebo (*p* = 0·05; Figure 4A). In contrast, fecal levels of TLR2 agonizts (PAM) and TLR5 agonizts (flagellin) did not changes significantly within or between groups at either time point (Figure 4B and C).

### Intestinal permeability

**Figure 5.**
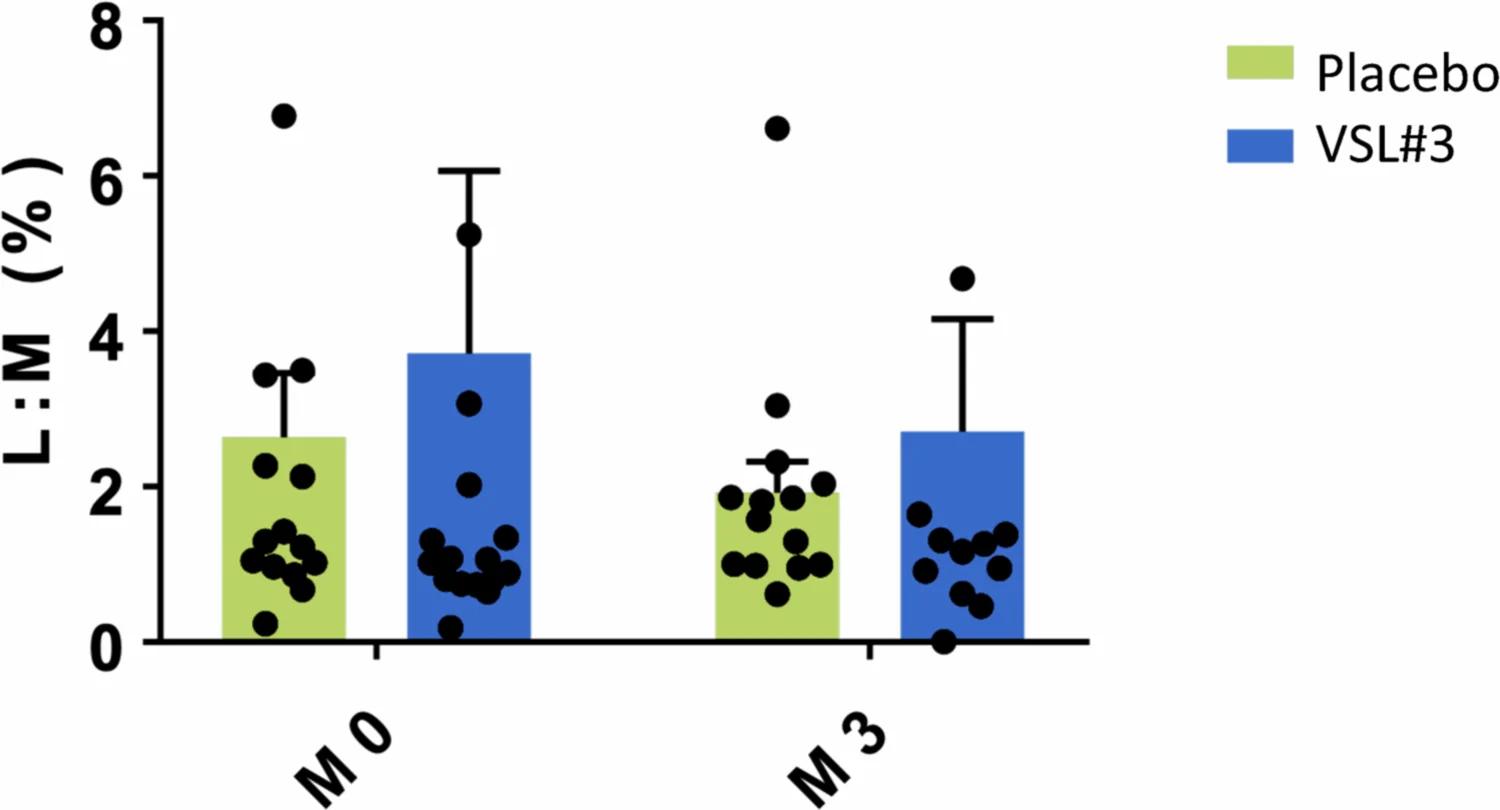
Intestinal permeability before and after treatment. Urinary lactulose-to-mannitol excretion ratio in patients at baseline (M0) and at month 3 (M3) according to treatment with VSL#3 or placebo. Data are presented as mean ± SD. Statistical analysis was performed using two-way ANOVA followed by Šídák's multiple comparisons test. Abbreviations: L: M (lactulose-to-mannitol excretion ratio); M0, baseline; M3, month 3; SD, standard deviation.

Intestinal permeability, assessed using the lactulose-mannitol (L/M) urinary excretion test, did not differ between groups (Figure 5). The L/M ratio remained below 7% in all patients, consistent with preserved intestinal barrier function.

### Gut microbiota composition according to fecal TLR4 agonist levels and ACR30 response

**Figure 6.**
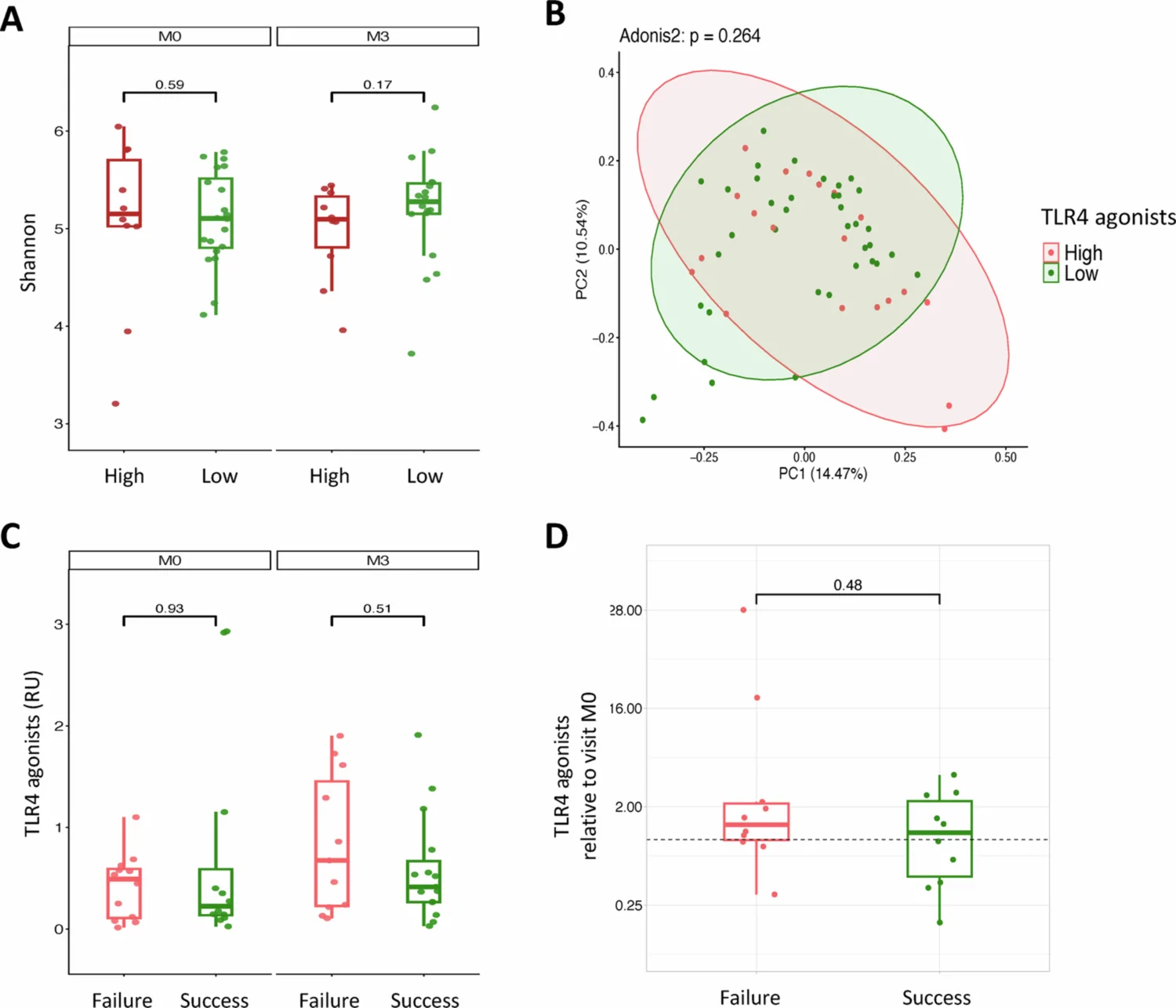
Gut microbiota composition according to fecal TLR4 agonist levels and ACR30 response. (A) Alpha diversity (Shannon index) of the stool microbiota at baseline (M0) and Month 3 (M3) according to high or low fecal TLR4 agonist (LPS) activity, defined using the median baseline fecal TLR4 agonist level as the cut-off. (B) Beta diversity (Bray–Curtis distance) of the stool microbiota at Month 3 (M3) according to high or low fecal TLR4 agonist (LPS) activity, defined using the median baseline fecal TLR4 agonist level as the cut-off; ellipses represent 95% confidence intervals. The *p* value corresponds to a PERMANOVA test performed using the adonis2 function from the vegan R package. (C) Fecal TLR4 agonist (LPS) activity according to ACR30 treatment response (responders vs. non-responders), measured using HEK-Blue reporter cells and expressed as relative units (RU). (D) Change in fecal TLR4 agonist (LPS) activity from baseline to Month 3 according to ACR30 treatment response (responders vs. non-responders), expressed as the M3/M0 ratio (relative to baseline [M0]).

No significant association were observed between fecal TLR4 agonizts levels and gut microbiota composition. Neither alpha diversity indices (Figure 6A) nor the relative abundance of major bacterial taxa (Figure 6B) were correlated with fecal TLR4 agonizts concentrations. Similarly, no association was found between ACR30 response and changes of fecal TLR4 agonist (Figure 6C and D).

### Serum cytokine and LPS-related marker profiles

Cytokine levels in serum are presented in Supplementary Figure 1. No significant differences were observed between groups at baseline or at M3. Similarly, no significant within-group changes were observed in the VSL#3 group between M0 to M3. In the post hoc exploratory analyzes, no significant differences in serum TLR4 agonist activity (used as a proxy for biologically active LPS) or anti-LPS IgG antibody levels were observed between treatment groups (Supplementary Figure 2).

## Discussion

This randomized controlled trial is, to our knowledge, the first to evaluate the clinical and microbial immunological effects of probiotic supplementation in children with oligoarticular or RF-negative polyarticular JIA. In this cohort, adjunctive treatment with VSL#3 did not improve disease activity after 3 months of treatment. Across prespecified ITT analyzes, treatment effects varied depending on assumptions regarding missing data, but no consistent evidence of benefit was observed. Notably, under conservative worst-case assumptions for missing data, response rates were numerically lower in the VSL#3 group, raising the possibility that probiotic supplementation may be neutral or even unfavorable in some patients.

The most notable mechanistic finding in this study was the significant increase in fecal Toll-like receptor 4 (TLR4) agonizts in the VSL#3 group. These agonizts are likely to include LPS, a key component of Gram-negative bacteria that can activate immune pathways via TLR4^11^ and has been shown to induce arthritis in murine models.^13^ Importantly, increased fecal TLR4 agonizts do not necessarily reflect expansion of Gram-negative taxa. Instead, they may arise from functional alterations in microbial activity, such as increased shedding of bacterial cell wall components or altered microbial turnover, occurring without detectable changes in overall microbiota composition.^4^ ^,^ ^25^ Probiotic-induced competition for nutrients or niche space may increase bacterial lysis or membrane permeability, resulting in greater luminal exposure to immunostimulatory microbial products. These findings suggest that probiotic supplementation may influence the functional output of the microbiota, particularly the release of microbial innate immune agonizts, even in the absence of measurable taxonomic restructuring. Such functional shifts in microbial output, independent of taxonomic restructuring, have been reported in other settings and provide a plausible explanation for the lack of clinical benefit observed here.^26^ These findings caution against assuming that probiotic supplementation is inherently anti-inflammatory and raise the possibility that, in some contexts, it may enhance exposure to pro-inflammatory microbial signals.

In contrast to the changes observed in fecal TLR4 agonizts, intestinal permeability remained within normal ranges throughout the study, and systemic cytokine levels did not change with VSL#3 supplementation. In addition, post hoc exploratory analyzes revealed no differences in serum LPS or anti-LPS antibody levels between treatment groups. Together, these findings suggest that the increased fecal TLR4 agonist activity was not accompanied by detectable systemic exposure to microbial products or systemic immune activation. However, the absence of differences in serum LPS-related markers does not exclude subtle or transient systemic translocation. Importantly, fecal TLR4 agonist activity reflects local gut luminal microbial immunostimulatory potential and should not be interpreted as a surrogate for systemic endotoxemia or clinical disease activity in this cohort. Alternative explanations for the increased fecal TLR4 agonist activity, including differences in the biological activity of microbial ligands or altered host responsiveness to TLR4 stimulation, cannot be excluded. The underlying mechanism and biological significance of this finding therefore remain incompletely understood and warrant further investigation.

However, the absence of a detectable systemic response in our cohort does not preclude local joint effects. In oligoarticular JIA, synovial inflammation may not be reflected in the serum cytokine levels, particularly for IL-6.^27^ Direct assessment of synovial fluid could clarify whether gut derived signals contribute to intra-articular immune activation, but such invasive sampling was not feasible in this young study population. Another contributing factor may be the limited capacity of oral probiotics to modify the gut microbiota. The intestinal microbiome is a highly resilient ecosystem in which established microbial communities exert strong competitive pressures for nutrients and ecological niches, limiting the ability of exogenously administered strains to engraft or induce durable compositional changes.^4^ ^,^ ^28^ Consistent with this, VSL#3 supplementation did not significantly alter gut microbiota diversity or composition in our cohort. In adults with inflammatory arthritis, probiotic trials have yielded inconsistent results, and many did not assess microbiota changes,^29-31^ further highlighting the challenges of translating probiotic supplementation into meaningful immunomodulation.

Our study focused on recent-onset juvenile idiopathic arthritis (JIA), with a median age of 4 y, with the aim of intervening during a period of greater microbiota–immune plasticity.^18^ However, as some host–microbiota interactions may have already been established by this age, short-term probiotic supplementation is likely to have only limited effects. These observations are consistent with broader evidence that brief interventions are generally insufficient to induce long-lasting changes in the composition of the gut microbiota or its functional inflammatory output.^4^ ^,^ ^32^ ^,^ ^33^ Durable modulation, particularly through sustained dietary changes, likely requires long-term adherence over months or years.^32^ ^,^ ^33^ It therefore remains possible that microbiome-targeted strategies may need to be initiated earlier in life—potentially during infancy or prior to disease onset—and maintained over longer periods to influence immune processes relevant to JIA. This hypothesis was not addressed in the present trial and should be considered exploratory. Taken together, the absence of clinical benefit after three months of VSL#3 supplementation may reflect the relative resilience of the gut ecosystem and a narrowing of the early-life 'window of opportunity' for durable microbiome modulation.

The effects of probiotics such as VSL#3 appear to be highly context dependent, reflecting complex immune activation signatures rather than uniform pro- or anti-inflammatory outcomes. Preclinical studies report mixed cytokine responses in human macrophages^27^ and dendritic cells,^34^ along with activation of epithelial NF‑κB,^16^ consistent with immune stimulation rather than simple suppression. In our study on JIA patients, VSL#3 increased fecal TLR4 agonizts without detectable changes in systemic cytokines or gut microbiota composition, indicating that probiotic supplementation may influence microbial immune signaling pathways without producing overt systemic inflammatory responses. Consistent with our results, a study of children with enthesistis-related arthritis reported no significant immunological or clinical benefit beyond NSAID therapy.^35^ Clinically studies have generally reported heterogenous effects of VSL#3 across inflammatory diseases, with no consistent evidence of efficacy across patient populations.^15^ Together, these findings indicate that probiotics cannot be assumed to be uniformly anti-inflammatory agents. Rather, their immunological effects appear to be dependent on host and disease-specific factors, highlighting the potential value of personalized strategies, potentially guided by baseline microbiota or immune profiling, to identify patients who might benefit from treatment or experience unintended immune activation.

No serious adverse events were observed in this trial. Nevertheless, rare but severe infectious complications related to probiotics use have been reported in immunocompromised patients.^36^ Given the uncertain immunomodulatory effects observed here, caution is warranted when considering probiotic supplementation in children with JIA. On the basis of these results, routine probiotic supplementation cannot be recommended for children with children with oligoarticular or RF-negative polyarticular JIA outside of clinical trials.

Strengths of this study include administration of probiotic treatment during early childhood, a period of dynamic microbiota development that may represent a sensitive window for intervention. The multicentre, randomized, double-blind, placebo-controlled design and the inclusion of complementary mechanistic assessments (intestinal permeability, gut microbiota composition, and fecal TLR agonizts) provide a robust evaluation of probiotic effects in children with oligoarticular or RF-negative polyarticular JIA.

The study also has several limitations. The trial was terminated before reaching the planned sample size because of slow recruitment. Consequently, the modest sample size and short follow-up reduced the power to detect modest treatment effects, increased the risk of type II error, and precluded assessment of longer-term outcomes owing to extensive missing data at later time points.

Therefore, although no significant clinical benefit of VSL#3 was observed, smaller clinically relevant treatment effects cannot be excluded. Furthermore, 68% of participants had absent or low disease activity at baseline, and received concomitant standard therapies, including NSAIDs and methotrexate. This may have introduced a floor effect, limiting the ability to detect an additional clinical effect of adjunctive probiotic treatment. Consequently, the observed absence of benefit—and under conservative assumptions, a signal towards worse outcomes in the probiotic group—should be interpreted cautiously, as the trial effectively assessed probiotics as an adjunct to established treatment in a population with low baseline inflammatory activity. Future studies may benefit from enrolling patients with a minimum disease activity threshold. The predominance of early-onset oligoarticular JIA may limit generalizability to other JIA subtypes. Moreover, a 3-month duration of probiotic supplementation may have been insufficient to induce sustained changes in the gut microbiota or to translate microbiome modulation into measurable clinical effects. Future studies should investigate whether longer treatment durations and/or interventions initiated earlier in life, when the gut microbiota is more plastic, result in greater microbiological and clinical responses. Furthermore, stool-based analyzes may not fully capture local gut or synovial immune processes. Although the study products were stored, transported, and handled according to the manufacturer's recommendations, and qualitative culture confirmed bacterial growth in the probiotic product, the viable bacterial counts throughout the study period could not be independently verified.

In conclusion, probiotic supplementation with VSL#3 did not improve disease activity in children with oligoarticular or RF-negative polyarticular JIA and was associated with changes in microbiota-derived innate immune signals of uncertain clinical significance. As the first multicenter, randomized, double-blind, placebo-controlled trial of probiotics in oligoarticular and RF-negative polyarticular JIA, this study provides important evidence to guide clinical decision-making in an area of widespread but poorly supported practice. Our findings highlight the complexity of host–microbiota interactions in JIA and suggest that future studies should explore personalized approaches to identify patients who might benefit—or be harmed—by probiotic interventions.

## Supplementary Material

Supplementary_Figures_and_tables.docx

CONSORT_checklist_PERMAJI.docx

## Data Availability

Individual participant data required to reach aims in an approved proposal, after de-identification, will be made available to investigators whose proposed use of the data has been approved by the study’s Executive Committee. The full trial protocol and statistical analysis plan are available from the corresponding author upon reasonable request. Proposals may be submitted up to 36 months after article publication and should be directed to the corresponding author.

## Role of the product supplier

The probiotic formulation (VSL#3) and corresponding placebo were kindly supplied by Laboratory ActialFarmaceutical, which had no involvement in the study design, data collection, data analysis, data interpretation, or manuscript preparation. The authors had full access to all study data and held final responsibility for the decision to submit the manuscript for publication.
