# Supplementary material for: Probiotic supplementation increases fecal TLR4 agonists without improving disease activity in juvenile idiopathic arthritis: a randomized placebo-controlled trial
Source: Gut Microbes. 2026 Sep 10;18(1):2728351. doi: 10.1080/19490976.2026.2728351 (PMC13568562; doi:10.1080/19490976.2026.2728351)
Supplement: Supplementary file 2 [file KGMI_A_2728351_SM1292.docx]

**Supplementary Table 1.**

Longitudinal changes in Bristol Stool Scale scores, nonsteroidal anti-inflammatory drug (NSAID) use, methotrexate use, and the occurrence of joint injections over the study period. Data are presented as median (interquartile range) for continuous variables and n (%) for categorical variables. NSAID, nonsteroidal anti-inflammatory drug.

| Variable | M0 | | | | M3 | |
| --- | --- | --- | --- | --- | --- | --- |
|  | | **VSL#3**  N=22 | | **Placebo**  N=22 | **VSL#3**  N=17 | **Placebo**  N=19 |
| Bristol stool chart  1  2  3  4  5  6  Médiane (Q1 ; Q3) | | | N=14 1 (7%) 7 (50%) 3 (21%) 0 (0%) 3 (21%) 0 (0%)  2,0 (2,0;3,0) | N=17 0 (0%) 8 (47%) 5 (29%) 0 (0%) 1 (6%) 3 (18%)  3,0 (2,0;3,0) | N=14 3 (21%) 3 (21%) 2 (14%) 3 (21%) 1 (7%) 2 (14%)  3,0 (2,0;4,0) | N=16 2 (13%) 5 (31%) 4 (25%) 5 (31%) 0 (0%) 0 (0%)  3,0 (2,0;4,0) |
| Treatments | | | | | | |
| NSAID   No  Yes | | | N=19 8 (42%) 11 (58%) | N=20 9 (45%) 11 (55%) | N=16 8 (50%) 8 (50%) | N=14 8 (57%) 6 (43%) |
| Methotrexate   No  Yes | | | N=20 14 (70%) 6 (30%) | N=20 14 (70%) 6 (30%) | N=16 10 (63%) 6 (38%) | N=13 9 (69%) 4 (31%) |
| Joint Infiltration between M0 and M3 | | | 0 (0%) | 0 (0%) | 1 (11%) | 2 (33%) |

**Supplementary Table 2.**

Details of all observed serious adverse events (SAEs) ans adverse events (AEs).

| **VSL#3** | **Placebo** |
| --- | --- |
| – | Hospitalization for left-sided pneumonia |
| **Total SAEs: 0** | **Total SAEs: 1** |
| Microcytic anemia | Growth and weight faltering under investigation |
| Bronchitis | Conjunctivitis |
| Diarrhea | Diarrhea |
| Post-traumatic ligament tear  of the right ankle | Acute gastroenteritis |
| Fever | Gastroenteritis |
| Fever | Influenza |
| COVID-19 infection | COVID-19 infection |
| Recurrence of knee and ankle pain | Acute otitis |
| Laryngeal cough | Acute otitis media |
| Anterior uveitis of the left eye | Anterior uveitis of the right eye |
| Vomiting and abdominal pain | Nasopharyngitis |
|  | Nasopharyngitis |
|  | Nasopharyngitis |
|  | Epistaxis (nosebleed) |
| **Total AEs: 11** | **Total AEs: 14** |

**Supplementary Table 3.**

Subgroup analysis of the primary endpoint according to age at inclusion (<3 vs. ≥3 years and <6 vs. ≥6 years), baseline nonsteroidal anti-inflammatory drug (NSAID) use, and baseline methotrexate use (with or without concomitant NSAID use). Comparisons between groups were performed using the χ² (chi-square) test. Data are presented as responders/evaluable patients, n (%). Abbreviations: ACR30, ≥30% improvement according to the American College of Rheumatology criteria; NSAID, nonsteroidal anti-inflammatory drug; ITT, intention-to-treat.

| ACR 30 - Primary endpoint | VSL#3  (N=22) | Placebo  (N=22) | p-value^[1]^ |
| --- | --- | --- | --- |
| ≤ 3 years (n=12) | N=6 | N=6 |  |
| 30% improvement without imputation at M3 | 1/5 (20%) | 4/6 (67%) | p=0.24 |
| 30% improvement with *best case* imputation at M3 | 2/6 (33%) | 4/6 (67%) | p=0.57 |
| 30% improvement with *worst case* imputation at M3 | 1/6 (17%) | 4/6 (67%) | p=0.24 |
| 30% improvement with *failure* imputation at M3 | 1/6 (17%) | 4/6 (67%) | p=0.24 |
| > 3 years (n=32) | N=16 | N=16 |  |
| 30% improvement without imputation at M3 | 7/12 (58%) | 8/13 (62%) | p>0.99 |
| 30% improvement with *best case* imputation at M3 | 11/16 (69%) | 8/16 (50%) | p=0.28 |
| 30% improvement with *worst case* imputation at M3 | 7/16 (44%) | 11/16 (69%) | p=0.15 |
| 30% improvement with *failure* imputation at M3 | 7/16 (44%) | 8/16 (50%) | p=0.72 |
| ≤ 6 years (n=33) | N=16 | N=17 |  |
| 30% improvement without imputation at M3 | 6/13 (46%) | 9/15 (60%) | p=0.46 |
| 30% improvement with *best case* imputation at M3 | 9/16 (56%) | 9/17 (53%) | p=0.85 |
| 30% improvement with *worst case* imputation at M3 | 6/16 (38%) | 11/17 (65%) | p=0.12 |
| 30% improvement with *failure* imputation at M3 | 6/16 (38%) | 9/17 (53%) | p=0.37 |
| > 6 years (=11) | N=6 | N=5 |  |
| 30% improvement without imputation at M3 | 2/4 (50%) | 3/4 (75%) | p>0.99 |
| 30% improvement with *best case* imputation at M3 | 4/6 (67%) | 3/5 (60%) | p>0.99 |
| 30% improvement with *worst case* imputation at M3 | 2/6 (33%) | 4/5 (80%) | p=0.24 |
| 30% improvement with *failure* imputation at M3 | 2/6 (33%) | 3/5 (60%) | p=0.57 |
| Methotrexate or Methotrexate + NSAID (n=14) | N=7 | N=7 |  |
| 30% improvement without imputation at M3 | 2/5 (40%) | 3/7 (43%) | p=0.44 |
| 30% improvement with *best case* imputation at M3 | 4/7 (57%) | 3/7 (43%) | p=0.36 |
| 30% improvement with *worst case* imputation at M3 | 2/7 (29%) | 3/7 (43%) | p=0.37 |
| 30% improvement with *failure* imputation at M3 | 2/7 (29%) | 3/7 (43%) | p=0.37 |
| NSAID (n=30) | N=15 | N=15 |  |
| 30% improvement without imputation at M3 | 6/12 (50%) | 9/12 (75%) | p=0.16 |
| 30% improvement with *best case* imputation at M3 | 9/15 (60%) | 9/15 (60%) | p>0.99 |
| 30% improvement with *worst case* imputation at M3 | 6/15 (40%) | 12/15 (80%) | **p=0.03** |
| 30% improvement with *failure* imputation at M3 | 6/15 (40%) | 9/15 (60%) | p=0.27 |

**Supplementary Table 4.**

Subgroup analysis of the primary endpoint according to baseline disease activity (none to low vs. moderate to high). Data are presented as n (%). Comparisons between groups were performed using the χ² (chi-square) test. Data are presented as responders/evaluable patients, n (%). Abbreviations: ACR30, ≥30% improvement according to the American College of Rheumatology criteria; ITT, intention-to-treat.

| ACR 30 - Primary endpoint | VSL#3 N=22 | Placebo N=22 | p-value^[2]^ |
| --- | --- | --- | --- |
| None to low disease activity (n=30) | N=15 | N=15 |  |
| 30% improvement without imputation at M3 | 6/11 (55%) | 10/13 (77%) | p=0.39 |
| 30% improvement with *best case* imputation at M3 | 10/15 (67%) | 10/15 (67%) | p>0.99 |
| 30% improvement with *worst case* imputation at M3 | 6/15 (40%) | 12/15 (80%) | **p=0.03** |
| 30% improvement with *failure* imputation at M3 | 6/15 (40%) | 10/15 (67%) | p=0.14 |
| Moderate to high disease activity (n=14) | N=7 | N=7 |  |
| 30% improvement without imputation at M3 | 2/6 (33%) | 2/6 (33%) | p>0.99 |
| 30% improvement with *best case* imputation at M3 | 3/7 (43%) | 2/7 (29%) | p=0.58 |
| 30% improvement with *worst case* imputation at M3 | 2/7 (29%) | 3/7 (43%) | p>0.99 |
| 30% improvement with *failure* imputation at M3 | 2/7 (29%) | 2/7 (29%) | p>0.99 |

**Supplementary Figure S1. Serum cytokine levels before and after treatment.**

Serum cytokine levels at baseline (M0) and month 3 (M3) according to treatment with VSL#3 or placebo. Each cytokine is shown in four panels with color coding indicated in the figure to represent treatment groups and timepoints (blue, placebo; green, VSL#3). Data are presented as boxplots showing median and interquartile range (Q1-Q3). *Abbreviations:* M0, baseline; M3, month 3; Q1, first quartile, Q3, third quartile.

**Supplementary figure S2: Serum TLR4 agonist activity and anti-LPS antibodies at baseline and 3 months after treatment.**

Serum TLR4 agonist activity, used as a surrogate measure of circulating LPS, and serum anti-LPS antibody levels were measured at baseline and after 3 months of treatment. Results are shown according to treatment group (VSL#3 or placebo). Data are expressed as fold change relative to the baseline (M0) placebo group.

**Supplementary Figure S3. Relative abundance of bacterial genera represented in the VSL#3 probiotic formulation.**

Relative abundance of bacterial genera included in the VSL#3 formulation at baseline (M0) and month 3 (M3) according to treatment with VSL#3 or placebo. Panels show: (A) *Streptococcus*, (B) *Bifidobacterium*, (C) *Lactobacillus*, (D) *Lactiplantibacillus*, (E) *Lacticaseibacillus*, and (F) combined relative abundance of all VSL#3-associated genera. Differential abundance analysis was performed using MaAsLin3 on genus-level relative abundance data, including treatment (VSL#3 vs placebo), timepoint (M0 vs M3), and their interaction as fixed effects, with participant included as a random effect. No significant treatment-related changes in the abundance of probiotic-associated genera were identified after correction for multiple testing. Although Streptococcus showed a trend toward increased abundance following VSL#3 supplementation, this did not reach statistical significance. Data are presented as relative abundance (%). Statistical significance was defined as an adjusted P value <0.05.
